# Multiple origins of BBCC allopolyploid species in the rice genus (*Oryza*)

**DOI:** 10.1038/srep14876

**Published:** 2015-10-13

**Authors:** Xin-Hui Zou, Yu-Su Du, Liang Tang, Xin-Wei Xu, Jeff J. Doyle, Tao Sang, Song Ge

**Affiliations:** 1 State Key Laboratory of Systematic and Evolutionary Botany, Institute of Botany, Chinese Academy of Sciences, Beijing, 100093, China; 2 University of Chinese Academy of Sciences, Beijing 100049, China; 3 School of Integrative Plant Biology, Plant Breeding & Genetics Section, Cornell University, Ithaca, NY 14853, USA

## Abstract

In the rice genus (*Oryza*), about one half of the species are allopolyploids. These species are not only important resources for rice breeding but also provide a unique opportunity for studying evolution of polyploid species. In the present study, we sequenced four biparentally inherited nuclear loci and three maternally inherited chloroplast fragments from all diploid and tetraploid species with the B- and C-genome types in this genus. We detected at least three independent origins of three BC-genome tetraploid species. Specifically, the diploid *O. punctata* (B-genome) and *O. officinalis* (C-genome) were the parental progenitors of *O. minuta* and *O. malampuzhaensis* with *O. punctata* being the maternal donors, whereas the diploid *O. punctata* and *O. eichingeri* (C-genome) were the progenitors of tetraploid *O. punctata* with *O. punctata* being the paternal donor. Our relaxed clock analyses suggest that all the BBCC species originated within the last one million years, which is coincident with the severe climate oscillations occurred during the last ice age, implying the potential impact of climate change on their formations and dispersals. In addition, our results support previous taxonomic arguments that the tetraploid *O. punctata* might be better treated as a separate species (*O. schweinfurthiana*).

Hybridization between diploid species associated with genome doubling to produce an allopolyploid species is a prevalent phenomenon in plant evolution^1^,^2^. Recent studies have demonstrated that allopolyploidization (interspecific hybridization and genome doubling) is one of the major modes of diversification and speciation in plants, and the important source of morphological innovations^3^. Many crop plants such as wheat, cotton, tobacco, and *Brassica* spp. (cabbage/rape) are of allopolyploid origins^4^. Thus, studying the origin of allopolyploids is not only the key to the understanding of consequences and mechanisms of polyploidization and crop domestication, but will facilitate also the genetic improvements for important crops and utilization of genetic resources in wild relatives of crop plants^1^,^4^.

As one of the major crops, cultivated rice belongs to *Oryza*, a medium-size genus that consists of two cultivated and about 20 wild species^5^,^6^. Because of its economic importance and tremendous genetic and genomic resources available, rice along with its wild relatives has become a unique model for both theoretical studies and practical crop improvement^7^,^8^,^9^. Particularly, with the completion of genome sequencing of two rice subspecies, the initiation and implementation of the *Oryza* Map Alignment Project (OMAP) have laid an important foundation for a complete genomic interrogation of the wild relatives of rice^7^.

Of 10 distinct genome types recognized for *Oryza* species, six are diploid (A, B, C, E, F and G) (2n = 2x = 24) and the other four are allotetraploid (BC, CD, HJ and HK) (2n = 4x = 48)^10^. Remarkably, about one half of the species in this genus are allotetraploids that originated through interspecific hybridization and genome doubling^6^,^7^,^11^. These allotetraploid species involved multiple independent polyploidizations, and include both ancient allotetraploids (HHJJ and HHKK) and recently formed ones (BBCC and CCDD)^7^,^10^,^11^, thus providing an increasingly attractive system for studies on the evolutionary dynamics of polyploid genomes organization and the mechanism of polyploidization in plants^12^,^13^,^14^. In addition, wild rice species including the polyploids have evolved under a wide range of climatic, geographic and ecological conditions over millions of years and conserved many useful alleles associated with yield-related traits and resistance to many abiotic and biotic stresses^6^. Previous studies showed that many valuable genes or alleles had been successfully transferred to cultivated rice from tetraploids, such as *O. minuta* (BBCC) and *O. grandiglumis* (CCDD), and have significantly increased rice yield and resistance to various diseases and pests^15^,^16^. Therefore, illustrating the origin and relationships of these tetraploids would also facilitate rice breeding and improvements.

To date, taxonomy and phylogeny of the rice genus have been extensively investigated and the evolutionary framework has been well established at the genus level^7^,^10^,^17^,^18^,^19^. Of the four tetraploid genomes, HJ and HK are ancient genomes and the diploid species that contributed the H, J, or K genomes have not been found (most likely extinct) despite great efforts. By contrast, two other tetraploid genomes (BC and CD) originated relatively recently and their parental diploid genomes/species are distributed widely across several continents^6^,^20^ (Fig. 1). Multiple lines of evidence have demonstrated that the CD genome (including three species) originated through a single allopolyploidization event with the C genome as the maternal parent^10^,^21^. In contrast, questions concerning the origin of the BC-genome species, including where, when and how the tetraploids originated have remained unanswered, although early preliminary studies suggested that the BC-genome species exhibited multiple origins or experienced introgression from sympatric diploid C-genome species^10^,^22^,^23^. These uncertainties have in turn caused confusion in taxonomy of the B-, C-, and BC-genome species^6^,^11^,^24^,^25^.

Based on several decades of field expeditions, at least three tetraploid species with the BC-genome type have been found so far^6^,^20^, two in Asia (*O. minuta* J.S. Presl. et C.B. Presl. and *O. malampuzhaensis* Krish. et Chand.) and the third (*O. punctata* Kotechy ex Steud.) in Africa (Fig. 1). In Asia, *O. minuta* is distributed in Philippines and Papua New Guinea, and *O. malampuzhaensis* has a localized distribution in South India near the town of Malampuzha^25^. For the diploid species, only a B-genome species (*O. punctata*) was found, which is widely distributed from east to west Africa (Fig. 1). Of three C-genome species, *O. officinalis* Wall. ex Watt is the most common species and is widely distributed in south China, South and Southeast Asia, and Papua New Guinea; whereas *O. rhizomatis* Vaughan has only been reported from Sri Lanka. The third C-genome species, *O. eichingeri* A. Peter, is disjunctively distributed in Sri Lanka and West and East Africa (Fig. 1)^20^,^26^. The overlapping geographical distribution and similarities in gross morphology between the diploid and tetraploid species lead further to the complexity of taxonomy and phylogeny of this group of species^6^,^11^.

Despite substantial attempts in previous studies on the B-, C-, and BC-genome species, most of them did not include all the diploid and tetraploid species in their studies^10^,^22^,^23^,^27^. Based on SSR and PCR-RFLP analyses, Bao *et al.* investigated the genetic diversity and species relationships by sampling all B-, C- and BC-genome species^28^,^29^. However, they were unable to obtain a fully resolved phylogeny due to limited genetic markers, and failed to identify the parental donors for the tetraploid species using only nuclear markers. In addition, the origin time of BBCC tetraploids in *Oryza* has been largely unknown with only *O. minuta* being dated previously^12^,^13^. Here, we sampled multiple populations from all the species involving the B-, C- and BC-genome types. Based on sequences of biparentally inherited nuclear genes and maternally inherited chloroplast regions, in conjunction with phylogenetic analyses and relaxed molecular dating, we fully resolved the phylogenetic relationships of these species, and particularly, determined the parental donors of all the BC-genome tetraploid species with their divergence times estimated. Specifically, we asked: (1) whether a single origin or multiple origins occurred for the BC-genome tetraploid species, (2) which diploid species were involved in the formation of the tetraploid species, and how many times did each contribute its genome, (3) when these allotetraploids originated. By illustrating the origin pattern of the BC-genome tetraploids in the rice genus, these investigations not only improve our understanding of evolutionary patterns of allopolyploid formation in the model system but also lay important foundation for utilization the wild rice germplasm in rice breeding and genetic improvement.

## Materials and Methods

### Plant Material and DNA sequencing

Twenty-two accessions representing all species consisting of the B- and C-genome types were sampled, including four diploid species (one B-genome and three C-genome species), and three tetraploid species with the BC-genome type. Note, two tetraploid *O. punctata* accessions were originally labeled as *O. eichingeri* in the germplasm bank but confirmed to be tetraploid *O. punctata*^27^,^29^,^30^. One accession of *Oryza granulata* with the G genome was used as an outgroup. Information on all the materials is listed in Table 1. Total DNA was isolated from fresh or silica-gel dried leaves using the cetyltrimethylammonium bromide method^31^.

We sequenced fragments of four single-copy nuclear genes that previously used in our phylogenetic studies of rice tribe Oryzeae, i.e., alcohol dehydrogenase-1 (*Adh1*), alcohol dehydrogenase-2 (*Adh2*), *leafy hull sterile 1* (*LHS1*), and heterotrimeric G protein (*GPA1*)^19^,^32^. Primers specific to these loci were reported in previous studies^10^,^32^. In the case when we failed to obtain the two homoeologs of the tetraploids, additional homoeolog-specific primers were designed. In addition, we chose to sequence three fast-evolving regions of the chloroplast genome based on Tang *et al.* (2010), including one chloroplast gene (*matK*) and two intergenic spacers (*rps16-trnQ* and *trnT-trnD*). All the primers and their sequences are listed in Supplementary Table S1. PCR amplifications were carried out by standard methods. The amplified PCR products were sequenced directly for all chloroplast fragments and for nuclear genes in case of diploid individuals. For all accessions of tetraploids, cleaned PCR products were cloned into pGEM T-easy vectors (Promega, Madison, WI, USA) and 12 to 20 clones per accession were selected for sequencing. Two types of clones (putative homoeologs) could be visually identified for each nuclear gene by using phylogenetic analyses of accession-specific clone sequences. A consensus sequence with multiple clones for each sequence type was used in the following phylogenetic analyses to minimize the effect of PCR errors. Sequencing was carried out on an ABI 3730 automated DNA sequencer (Applied Biosystems, Foster City, CA, USA). All DNA sequences used for this study have been deposited in the GenBank database under accession numbers KP121693 - KP121896, and the sequence alignments have been uploaded to TreeBASE (Accession URL: http://purl.org/phylo/treebase/phylows/study/TB2:S18041).

### Phylogenetic analyses

Low-copy nuclear genes in combination with chloroplast sequences have proven to be a very effective way to address allopolyploidization event at the species level^10^,^21^,^33^. Because the homoeologous sequences of nuclear loci in allopolyploids are contributed by both of the diploid parents while chloroplast genome is maternally inherited in most angiosperms including species in the rice genus, the combined analyses of biparentally inherited nuclear genes and maternally inherited chloroplast regions enable us to identify parental donors of allotetraploids^10^,^21^,^33^. In this study, both chloroplast and nuclear sequences were aligned using MUSCLE^34^ and then manually adjusted, with the regions of ambiguous alignment excluded. Prior to phylogenetic analyses, nucleotide frequencies were assessed for deviation from stationarity with the Chi-square test and no significant heterogeneity of base frequencies (P = 1.00) was detected in our data. To identify potential intragenic recombinants, the RDP program were used to examine the alignments, with six recombination detection methods (RDP, GENECONV, Chimaera, MaxChi, BootScan and SiScan) implemented and the default settings used^35^. The recombinant sequences identified by RDP program were excluded from the phylogenetic analyses.

Phylogenetic trees were reconstructed using the maximum parsimony (MP) and maximum likelihood (ML) methods with PAUP* 4.0b10 36. For MP analysis, tree searches were performed using heuristic searches with tree bisection and reconnection (TBR) branch swapping and 1000 replicates of random addition sequence, with Multrees option on. Statistical reliability of topology was estimated by bootstrap analyses with 1000 replicates. For ML analysis, the best-fit model of DNA substitution was selected by the Akaike information criterion (AIC) in jModelTest 2^37^. Tree searches were performed using the heuristic algorithm and support for clades was assessed by bootstrap analyses with 500 replicates. The three chloroplast regions were concatenated for the phylogenetic analysis since they are genetically linked and form a single historical and phylogenetic unit. For the four nuclear loci, phylogenetic analyses were conducted on individual genes separately. After evaluating single gene trees, two distinct sequence types corresponding to two homoeologs could be identified for each tetraploid. Then, homoeologous sequences of four loci were concatenated and analyzed using ML and MP methods.

Besides the analyses of single-gene and concatenated sequences, we inferred the species trees using the four nuclear data sets using *BEAST^38^, which is based on coalescent models. The 22 accessions of ingroup, including two homoeologues for each allotetraploids, were grouped into 10 operational taxonomic units (OTUs). The MCMC runs were set to 100 million generations, taking samples every 10000 generations, and convergence of MCMC chain was checked by running at least two independent analyses and by Tracer v1.4.

### Estimation of divergence time

We estimate the divergence times in the relaxed-clock framework using two commonly used Bayesian Markov chain Monte Carlo (MCMC) programs, BEAST v1.7.0^39^ and MCMCTREE in PAML v4.8^40^. These two relaxed-clock methods can account for the rate heterogeneity across lineages and accommodate multiple calibrations. Moreover, they can incorporate multiple loci into one analysis and deal with the different rates among loci appropriately.

In BEAST analyses, we used a relaxed clock model with the rate for each branch drawn from lognormal distribution. No topological constraints were used and we chose the coalescent as the tree prior since the phylogeny under scrutiny is a gene phylogeny rather than a species phylogeny^39^,^41^. The best-fit model of DNA substitution was used for each locus as selected by jModelTest 2^37^. Our previous estimates based on 106 single-copy nuclear genes^18^ indicated that the G-genome species originated about 13.5 ~ 15.5 million years ago (Ma) and the C-genome species diverged from the lineage consisting of the A- and B-genome species around 5.5 ~ 6.5 Ma. Accordingly, we calibrated the divergence of G-genome with a normally distributed prior having a mean of 14.5 Ma and a standard deviation (SD) of 1.5 Ma, and the divergence of C-genome with a normally distributed prior having a mean of 6 Ma and a SD of 1 Ma. Final analyses consisted of 20 million generations of MCMC runs with sampling every 2000 generations and the initial 20% samples as burn-in.

In MCMCTREE analyses, soft bounds are imposed so that the minimum and maximum age constraint may be violated with a small probability (2.5%). We used the HKY85+Γ substitution model with different transition/transversion rate ration parameter (κ) and different shape parameter (α) among loci, and the two calibration points were set similarly to the BEAST analyses. The ML tree obtained from the concatenated analyses was used as the input tree. A total of 100,000 generations was run with sampling every five generations after discarding the initial 10,000 samples as burn-in. In all dating analyses, each run was conducted at least twice to ensure consistency between different runs and convergence of the MCMC was evaluated by Tracer v1.4^42^.

## Results

Through PCR-amplifying, cloning and sequencing, we identified two types of distinct sequences at four single-copy nuclear loci for all allotetraploid accessions, corresponding to homoeologs of the B and C genomes, respectively. All four genes recovered similar gene trees in terms of topology, with two major clades (Supplementary Fig. S1). The first clade consisted of the accessions of all diploid B-genome species and the B-genome homoeologs of the tetraploid species, while the other clade included accessions of all diploid C-genome species and the C-genome homoeologs of the tetraploids (hereafter named as the clade B and clade C, respectively). The bootstrap values for the two major clades were all 100% except for *GPA1* in which the bootstrap value for clade C was over 90%.

**Figure 2:**
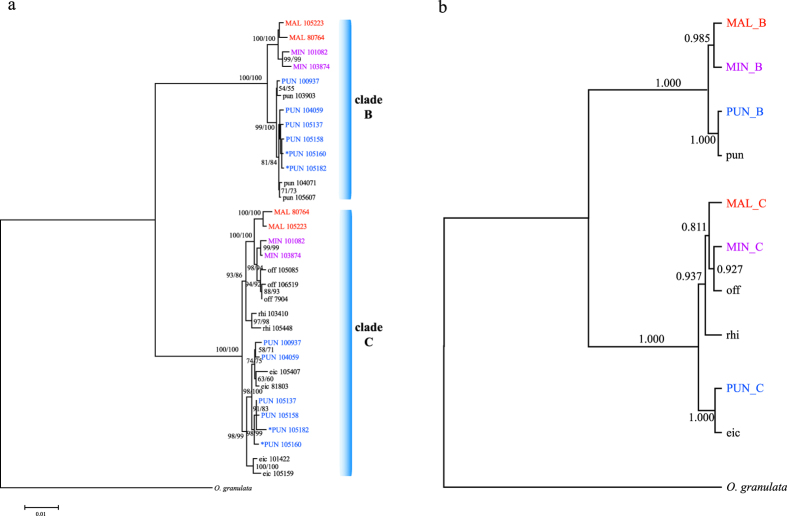
(**a**) ML tree of the *Oryza* species with the B-, C- and BC-genome types based on the concatenated nuclear gene data. Two main clades represent homoeologous groups with the B and C genomes, respectively. Numbers besides nodes are ML/MP bootstrapping support over 50%. (**b**) Species tree inferred using *BEAST with posterior probability indicated besides the branches. Sample abbreviation is indicated in Table 1, with tetraploids labeled with colored capital letters.

Because the topology within the major clades was less resolved in individual gene trees due to insufficient informative sites, and given the substantial congruence among the four gene trees, we conducted a combined analysis based on the concatenated sequences from the four nuclear loci, with the variable sites being 16.1% and informative sites 7.3%. The resulting phylogenetic trees inferred from ML and MP methods were the same and well resolved (Fig. 2a). Within both major clades, two Asian tetraploids (*O. minuta* and *O. malampuzhaensis*) and the African tetraploid *O. punctata* were grouped into two clearly separate subclades with 93–100% ML bootstrap support. Within clade B, accessions from the two Asian tetraploids formed a monophyletic group with 100% support and those from African tetraploids grouped with the diploid *O. punctata* (BB) with 99–100% support. Within clade C, accessions from the two Asian tetraploids grouped with *O. officinalis* with 100% support, with this clade sister to *O. rhizomatis*, while accessions from African tetraploids grouped with *O. eichingeri* with 98–99% support. These results indicated that, of three C-genome diploid species, *O*. *officinalis* was most likely involved in the formation of two Asian tetraploid species while *O. eichingeri* was most likely the C-genome donor of the African tetraploid species (Fig. 2a).

In addition to the individual gene analyses and concatenated analyses, we inferred the species trees using *BEAST which utilized information contained in individual nuclear loci based on coalescent models (Fig. 2b). Each of the homoeologous genomes of each tetraploid species was grouped with its putative diploid progenitor and the overall topology corroborated the concatenated tree with good support. Based on these analyses, it could be inferred that diploid *O. punctata* and *O. officinalis* were most likely the parental progenitors of *O. minuta* and *O. malampuzhaensis*, whereas the diploid *O. punctata* and *O. eichingeri* were the progenitors of tetraploid *O. punctata*. Note that two tetraploid *O. punctata* accessions marked with asterisks in Fig. 2a and Supplementary Fig. S1 were always grouped with accessions from other tetraploid *O. punctata* in both concatenated and single-gene analyses, supporting the previous treatment of these two accessions as tetraploid *O. punctata* rather than *O. eichingeri*^27^,^30^.

**Figure 3:**
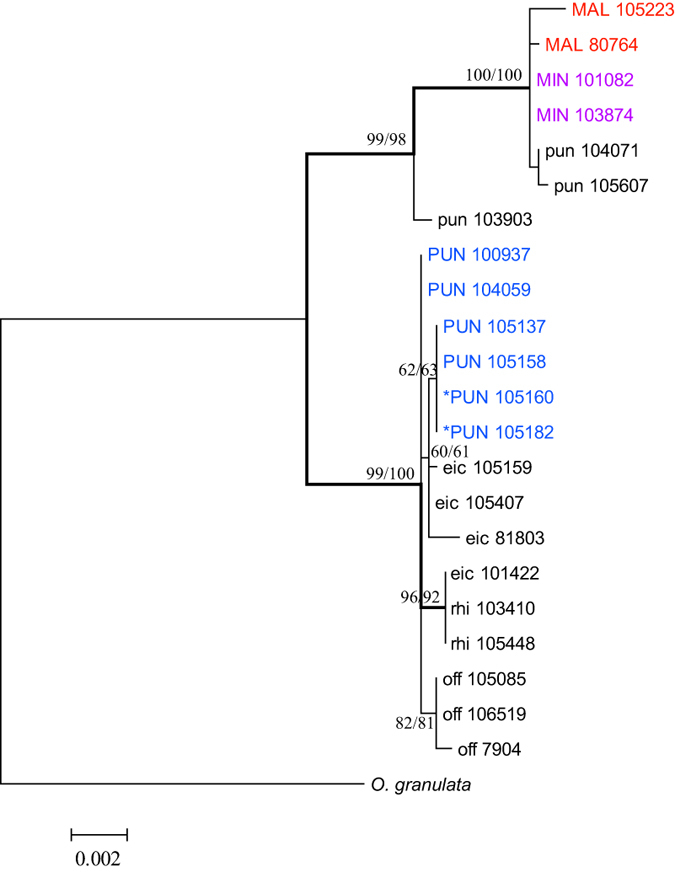
ML tree based on the concatenated chloroplast sequences. Numbers besides nodes are ML/MP bootstrapping supports over 50%. Branches having more than 90% ML and MP bootstrap supports are indicated by thickened lines.

The concatenated three chloroplast regions produced a haplotype dataset of 3713 bp in length, with 3.6% variable sites and 1.2% informative sites. The inferred phylogeny recovered a 100% supported monophyletic group consisting of haplotypes from tetraploid *O. minuta* and *O. malampuzhaensis* and diploid *O. punctata*, supporting the latter as the maternal donor of Asian tetraploids (Fig. 3). In the other clade, haplotypes from four out of six African tetraploid accessions grouped with diploid *O. eichingeri* haplotypes (with support of 60–61%), while another two were not resolved due to the lack of phylogenetic information. In combination with the results from the nuclear gene tree, this corroborated the hypothesis that *O. eichingeri* was most probably the maternal genome donor of the African tetraploids. Interestingly, alleles or haplotypes from individuals of tetraploid *O. punctata* did not form a monophyletic clade in all nuclear and chloroplast gene trees, implying recurrent origins of the tetraploid *O. punctata*.

**Figure 4:**
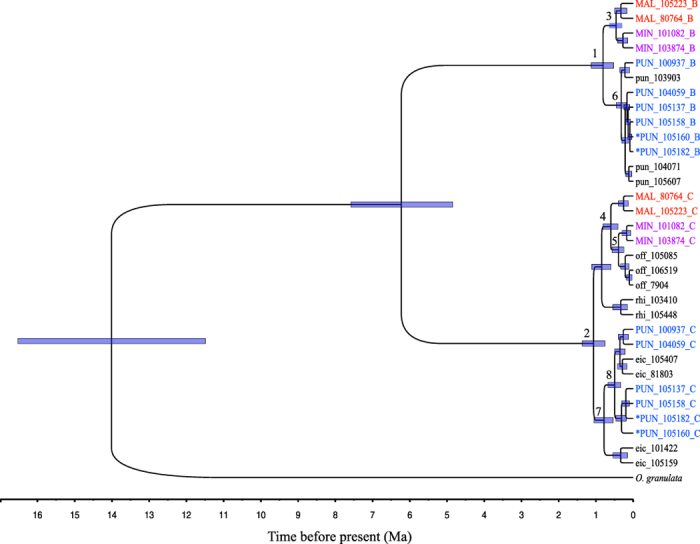
Chronogram of the *Oryza* species with the B-, C- and BC-genome types obtained based on four nuclear loci using relaxed clock implemented in BEAST. Branch lengths indicate the posterior means of date estimates with the blue bars representing 95% highest posterior density (HPD) intervals for the divergence times estimates. Nodes of interest are numbered.

We estimated the time of origin of BBCC tetraploids by utilizing relaxed clock methods based on sequences of nuclear markers. The Bayesian analyses using BEAST resulted in a completely congruent phylogeny to that from concatenated analyses. As shown in Fig. 4, nodes 6 and 8 are both potential nodes of origin of African tetraploids, with each being the divergence time of one of the homoeologs from their diploid progenitors respectively. Considering the standing variation of alleles in diploid progenitors, the youngest estimate, or the minimum distances between alleles of a tetraploid and that of its diploid progenitor are typically assumed to be the origin time of the tetraploid^43^. Therefore, the tetraploid *O. punctata* originated about 0.19–0.46 Ma (node 6) and *O. minuta* originated slightly earlier (about 0.25–0.57 Ma) (node 5). It is difficult to estimate the specific time of origin of *O. malampuzhaensis*, since all individuals did not closely group with any diploids. Because the C copy of *O. minuta* first grouped with its diploid donor rather than *O. malampuzhaensis*, it is most likely that the two Asian tetraploids originated independently. Therefore, we speculate that the origin of *O. malampuzhaensis* might be more recent than the date of the nodes 3 and 4, (later than 0.81 Ma). The estimates of the age of tetraploid origin using MCMCTREE resulted in similar results and the 95% HPD intervals of key nodes overlapped considerably with those using BEAST (Supplementary Fig. S2 and Table S2). Given the estimates from the two relaxed clock methods, it appears that all BBCC species in *Oryza* originated within the last 1 million years.

## Discussion

**Figure 5:**
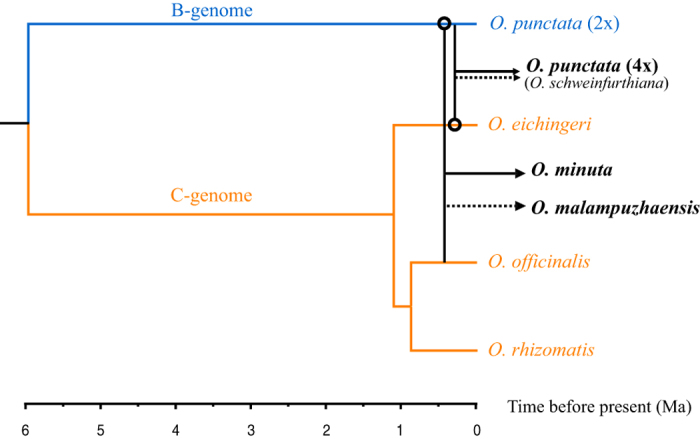
Schematic of evolutionary relationships among the species with the B-, C-, and BC-genome types, with emphasis on the origins of the allotetraploid species. Black lines represent the origins of the allotetraploids with the maternal donors indicated by circles. The solid and broken arrows indicate the confirmed and hypothetical allopolyploidization events, respectively.

The *Oryza* species with the B-, C- and BC-genome types have long been a subject of debate for both species delimitation and species relationships^6^,^11^,^24^,^25^. For example, both diploid and tetraploid forms of *O. punctata* were found in Africa, with distinct morphological differences between them^44^,^45^,^46^. Therefore, some taxonomists used *Oryza schweinfurthiana* Prod. to refer to the tetraploid *O. punctata*^47^. However, this treatment was not accepted by other authors. Similarly, the tetraploid *O. eichingeri* has been reported before^44^, but was considered to be a misidentification of tetraploid *O. punctata* when its morphology^25^,^46^ and genetic markers^27^,^28^,^30^ were analyzed. Another confounding issue is the identities of the exact parental donors of each allotetraploid, which have been inconsistent among previous studies. For example, in the case of *O. malampuzhaensis*, *O. officinalis* was indicated as its C-genome progenitor based on a study uisng AFLP markers^27^ while either *O. rhizomatis* or *O. eichingeri* was regarded as the C-genome donors based on nuclear RFLP and SSR markers^23^,^29^. In the present study, we generated a well-resolved phylogenetic tree of all the diploid and tetraploid species with the B- and C-genome types based on sequences from biparentally inherited nuclear genes and maternally inherited chloroplast fragments. The main findings of this study are illustrated by Fig. 5 and have several important implications. First, we demonstrated that at least three independent allotetraploid events gave rise to three extant BC-tetraploid species in this species complex, and the tetraploid *O. punctata* (*Oryza schweinfurthiana*) appears to have originated multiple times. Multiple origins were detected despite the fact that only a small number of accessions was sampled (Fig. 2). Interestingly, the single B-genome diploid species, *O. punctata*, served as the maternal progenitor for two Asian tetraploids (*O. minuta* and *O. malampuzhaensis*) and as the paternal progenitor for the African tetraploid (*O. punctata* or *O. schweinfurthiana*) (Fig. 5). Similar pattern of multiple origins or reticulate evolution of polyploidy species have been well documented in many model systems such as *Tragopogon*, *Mimulus*, *Rubus*, and *Glycine* (reviewed by Soltis *et al.*, 2014). With enormous genetic, genomic and functional resources available^7^,^9^, the *Oryza* species can become an additional model for investigations on polyploid speciation and evolution.

Second, we clearly identified the most likely parental diploid species that contributed to the formation of two allotetraploid species. Specifically, *O. minuta* appears to have originated from allopolyploidization of *O. punctata* (maternal) and *O. officinalis* (paternal); whereas the tetraploid *O. punctata* (*O. schweinfurthiana*) was derived from *O. eichingeri* (maternal) and *O. punctata* (paternal) (Fig. 5). Although it is evident that the maternal and paternal donors of *O*. *malampuzhaensis* are the B-genome and C-genome species, respectively, the exact diploid C-genome species involving the tetraploid formation remains to be identified (Figs 2 and 5). Of three C-genome diploid species, *O. rhizomatis* was not involved in the formation of extant polyploids. These findings raise some interesting questions regarding where and when the allopolyploidization events have taken place for the two Asian tetraploids, given that the distribution of extant diploid *O. punctata* is confined to Africa. Did the polyploidization occur in Africa and then disperse to Asia? Alternatively, the events could have taken place in Asia with the diploid B-genome donor later becoming extinct. The situation is reminiscent of *Gossypium*, where the New World allopolyploid *G. hirsutum* combines a New World D-genome with an exclusively African A-genome^48^. The clarification of the reticulate relationships of the *Oryza* species with the B- and C-genome types will inevitably facilitate the investigations of polyploid evolution using this group of species as working system given the fact that the *Oryza* polyploids have been increasingly used for studying polyploidization and genome evolution^7^,^12^,^13^.

Third, our phylogenetic results provided valuable information to guide taxonomic treatment of the tetraploids. Unlike *O. minuta* and *O. malampuzhaensis* that have been proved good species^27^,^28^,^49^,^50^, the debate over the delimitation of the African BC-genome tetraploids has continued for decades^20^,^46^. Earlier studies have claimed that both diploid and tetraploid forms were found for *O. punctata* and *O. eichingeri*^44^,^46^ and the tetraploid *O. punctata* was distributed as widely as its diploid form^28^. Because the diploid and tetraploid forms of *O. punctata* differed by annual and perennial habits and in many morphological characteristics^45^, the name “*Oryza schweinfurthiana* Prod.” has been used by some taxonomists to refer to the tetraploid *O. punctata*^47^. Our study revealed that the tetraploid *O. punctata* arose by hybridization and polyploidization of two distinct diploid species (*O. punctata* and *O. eichingeri*); along with distinct gross morphology and life history, this strongly supports the species status of the tetraploid form of *O. punctata*, i.e., *O. schweinfurthiana*. With regard to the tetraploid *O. eichingeri*, Tateoka (1965) pointed out the possibility of misidentification. Several investigations based on molecular markers have also demonstrated that some of the materials designated as tetraploid *O. eichingeri* were instead tetraploid *O. punctata*^27^,^29^,^30^. In this study, homoeologues from two tetraploid *O. eichingeri* accessions grouped with tetraploid *O. punctata* rather than forming a monophyletic clade in all cases (Figs 2 and 3, Supplementary Fig. S1), providing further evidence that *O. eichingeri* is a diploid species and the materials previously identified as tetraploid *O. eichingeri* are most likely to be the tetraploid *O. punctata.*

Finally, through relaxed molecular clock analyses, we estimated that the BC-genome tetraploid species arose around 0.2–0.6 Ma (Figs 4 and 5), largely consistent with previous studies^12^,^13^. These estimates suggest that the BC-genome tetraploid species originated recently relative to the divergence of the A, B, and C genomes, around 5.5 ~ 6.5 Ma^18^. It is interesting to ask what factors may have contributed to the formation of the BC-genome tetraploids given the fact that no any polyploid species have been found among the eight A-genome species. It is well recognized that evolution of earth’s terrestrial biota is profoundly influenced by the global climate changes^51^,^52^. Evidence showed that there was a period of dramatic climate change within the series of ice ages from 0.9 Myr to the last ice age, during which severe climate oscillations happened followed a 100-Kyr cycle and average temperature would rise rapidly by about 7 °C over just decades^53^. The origin of the BC-genome tetraploids occurred coincidentally with this period, implying the potential impact of climate change on the formation and dispersal of these tetraploids. Recent studies suggest that the polyploidy events may have generated sufficient novelty that increased tolerance toward the drastically changing global environment and thus enhanced the adaptability of species^2^,^54^. This is consistent with the observed correlation between polyploidy and invasiveness^55^.

It should be noted, nevertheless, that the materials we used in this study are limited in term of sample sizes per species, because of the difficulty in obtaining samples for this group of species. Therefore, the origin times of polyploids we estimated here is the maximum, and more recent dates could have been obtained by additional sampling^43^. Such sampling schemes also preclude an in-depth investigation of the potential diploid progenitors of some tetraploids (e.g., *O. malampuzhaensis*) and exact place of polyploid origin and their subsequent dispersals, as well as the potential populations that contributed to the multiple formations of specific tetraploid species (e.g., the tetraploid *O. punctata* or *O. schweinfurthiana*). Further studies based on more extensive sampling across the entire distribution areas of the BC-genome species are needed to elucidate the evolutionary scenario of this species complex.

## Additional Information

**How to cite this article**: Zou, X.-H. *et al.* Multiple origins of BBCC allopolyploid species in the rice genus (*Oryza*). *Sci. Rep.* **5**, 14876; doi: 10.1038/srep14876 (2015).

## Supplementary Material

Supplementary Information

## Figures and Tables

**Figure 1:**
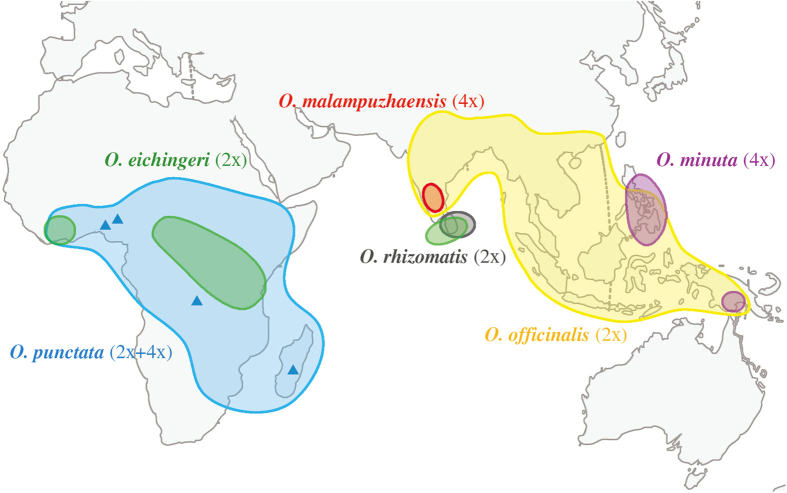
Geographical distribution of the *Oryza* species with the B-, C- and BC-genome types using software Adobe PhotoShop (Data from Vaughan 1994 and 1989)^6^,^20^. Blue, diploid *O. punctata* with the tetraploid *O. punctata* indicated by triangles; Yellow, *O. officinalis*; Green, *O. eichingeri*; Grey, *O. rhizomatis*; Purple, *O. minuta*; Red, *O. malampuzhaensis*.

**Table 1:** Comprehensive list of the samples used in the present study including species name, genome type, source, abbreviation in figures, and accession numbers in the International Rice Research Institute (IRRI).

Species	Genome types	Source	Accession	Abbreviation
*O. punctata*	BB	Tanzania	103903	pun_103903
*O. punctata*	BB	Cameroon	104071	pun_104071
*O. punctata*	BB	Chad	105607	pun_105607
*O. eichingeri*	CC	Uganda	101422	eic_101422
*O. eichingeri*	CC	Uganda	105159	eic_105159
*O. eichingeri*	CC	Sri Lanka	105407	eic_105407
*O. eichingeri*	CC	Sri Lanka	81803	eic_81803
*O. officinalis*	CC	Philippines	105085	off_105085
*O. officinalis*	CC	PNG	106519	off_106519
*O. officinalis*	CC	China	7904	off_7904
*O. rhizomatis*	CC	Sri Lanka	103410	rhi_103410
*O. rhizomatis*	CC	Sri Lanka	105448	rhi_105448
*O. malampuzhaensis*	BBCC	India	105223	MAL_105223
*O. malampuzhaensis*	BBCC	India	80764	MAL_80764
*O. minuta*	BBCC	Philippines	101082	MIN_101082
*O. minuta*	BBCC	Philippines	103874	MIN_103874
*O. punctata*	BBCC	Ghana	100937	PUN_100937
*O. punctata*	BBCC	Nigeria	104059	PUN_104059
*O. punctata*	BBCC	Zaire	105137	PUN_105137
*O. punctata*	BBCC	Kenya	105158	PUN_105158
*O. punctata	BBCC	Uganda	105160	*PUN_105160
*O. punctata	BBCC	Uganda	105182	*PUN_105182
*O. granulata*	GG	Vietnam	106469	*O. granulata*

All accessions were obtained from leaf materials or seeds provided by IRRI at Los Banos, Philippines, except for 7904, which was collected by the authors.

Two accessions marked with asterisks were originally labeled as *O. eichingeri* in the Germplasm Resource Center of IRRI but confirmed to be tetraploid *O. punctata*^27^,^29^,^30^.
