# Supplementary material for: Multiple origins of BBCC allopolyploid species in the rice genus (Oryza)
Source: Sci Rep. 2015 Oct 13;5:14876. doi: 10.1038/srep14876 (PMC4602239; doi:10.1038/srep14876)
Supplement: Supplementary Information [file srep14876-s1.pdf]

Supplementary information

Multiple origins of BBCC allopolyploid species in the rice genus (*Oryza*)

Xin-Hui Zou[1], Du-Yu Su[1,2], Liang Tang[1,3], Xin-Wei Xu[1,4], Jeff J. Doyle[5], Tao Sang[1], Song Ge[1,2,*]

Supplementary Figure S1. Individual gene trees inferred from ML analysis of four nuclear genes. The gene name of each locus is indicated below the tree. Numbers besides branches are ML/MP bootstrapping support with values lower than 50% not shown. Accessions are represented by abbreviations as shown in table 1, and the tetraploids are labeled with colored and capital letters.

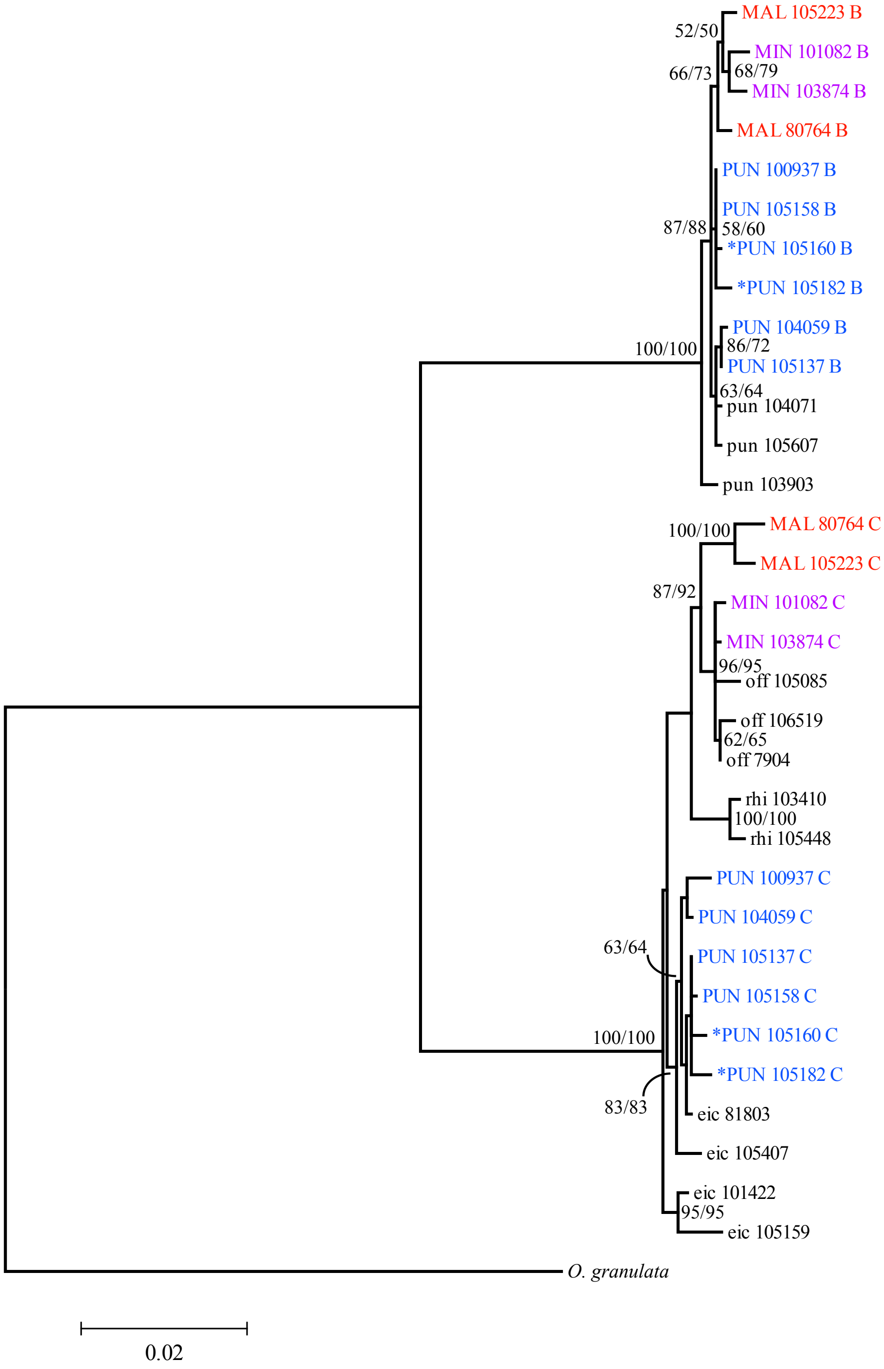

Adh1

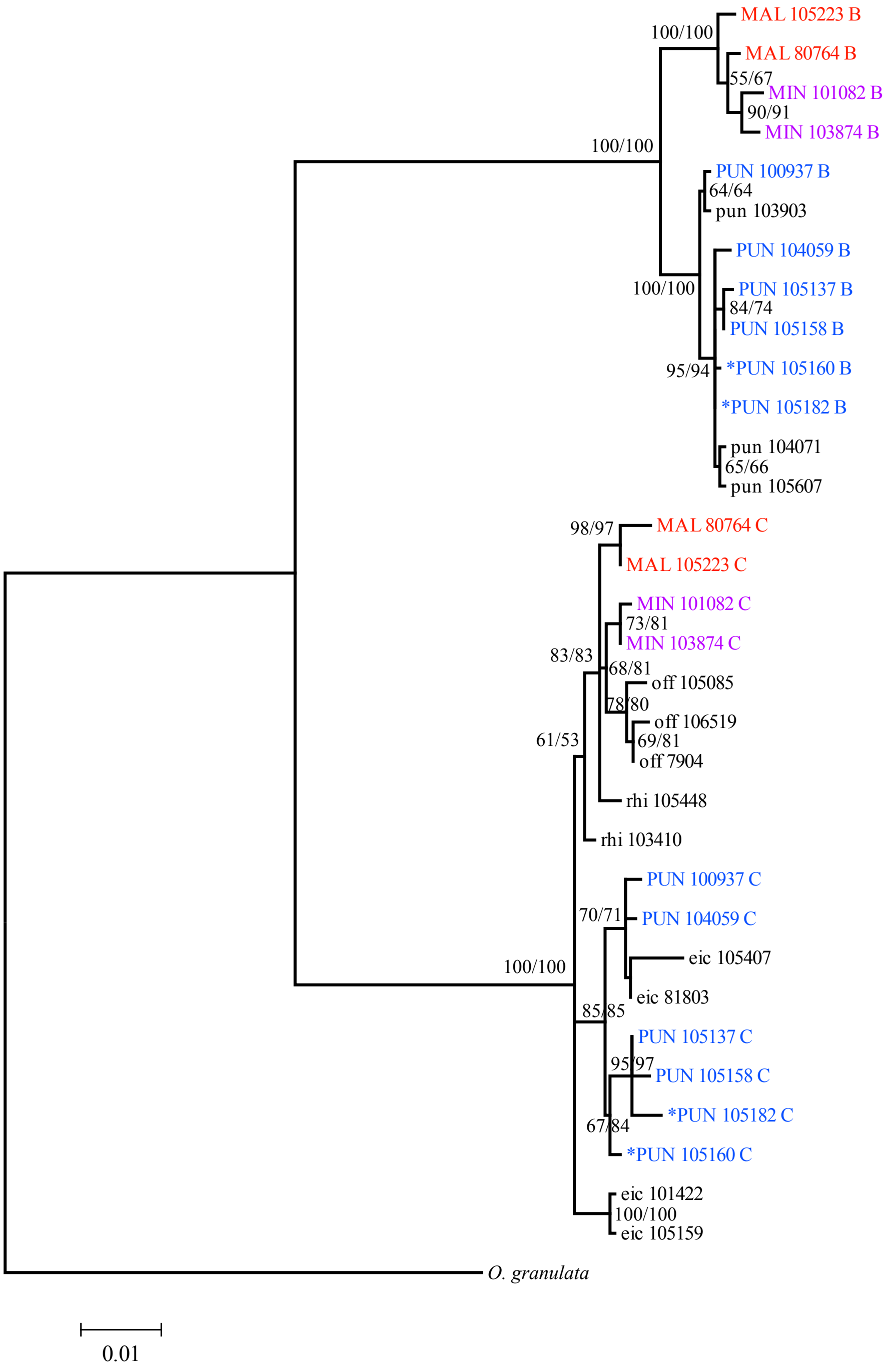

MAL 105223 B
100/100
MAL 80764 B
55/67
MIN 101082 B
90/91
MIN 103874 B
100/100
PUN 100937 B
64/64
pun 103903
PUN 104059 B
100/100
PUN 105137 B
84/74
PUN 105158 B
95/94
*PUN 105160 B
*PUN 105182 B
pun 104071
65/66
pun 105607
98/97
MAL 80764 C
MAL 105223 C
MIN 101082 C
73/81
MIN 103874 C
83/83
68/81
off 105085
78/80
off 106519
61/53
69/81
off 7904
rhi 105448
rhi 103410
PUN 100937 C
70/71
PUN 104059 C
eic 105407
100/100
eic 81803
85/85
PUN 105137 C
95/97
PUN 105158 C
*PUN 105182 C
67/84
*PUN 105160 C
eic 101422
100/100
eic 105159
O. granulata
0.01

Adh2

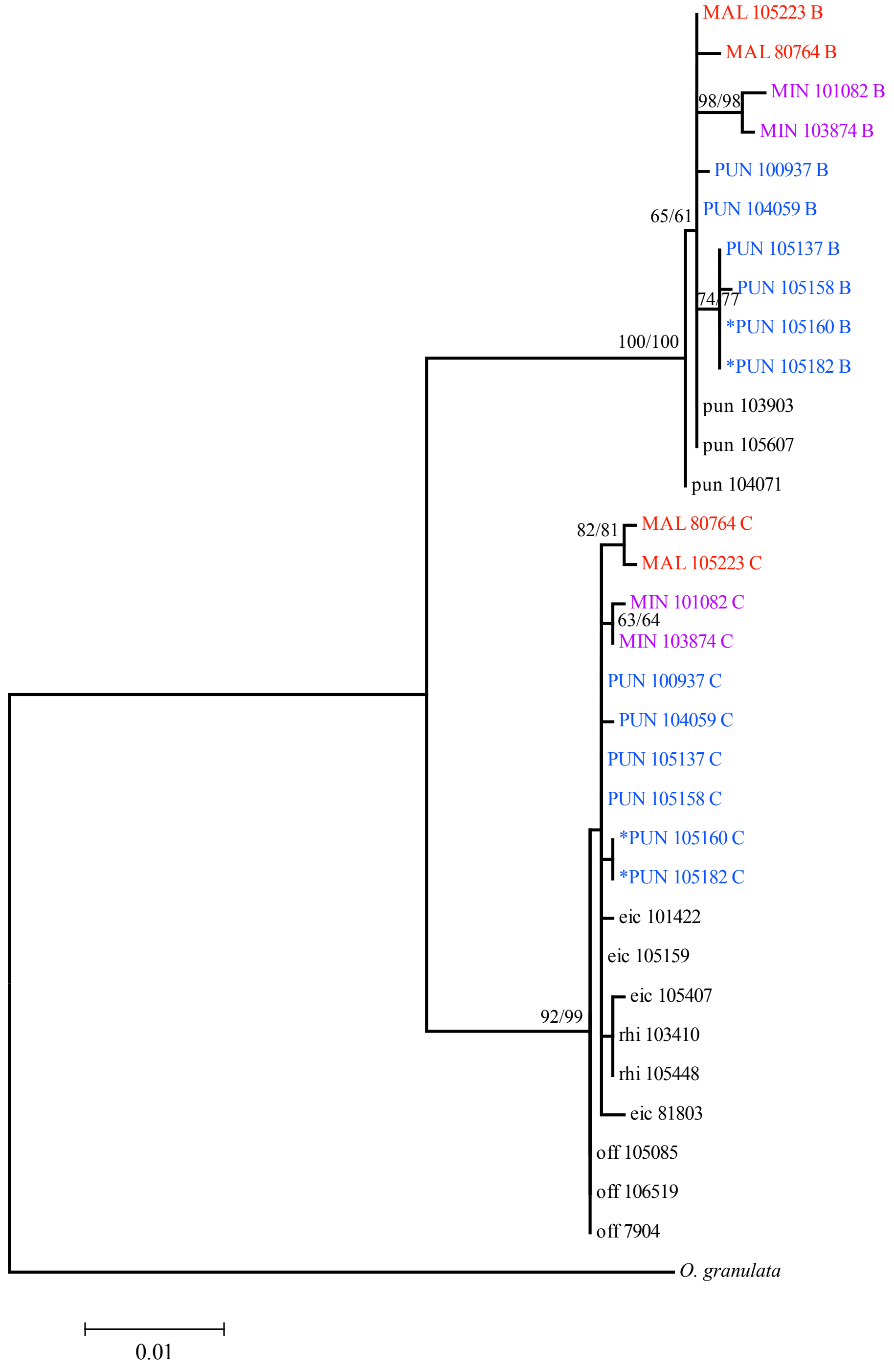
MAL 105223 B
MAL 80764 B
98/98
MIN 101082 B
MIN 103874 B
PUN 100937 B
65/61
PUN 104059 B
PUN 105137 B
PUN 105158 B
74/77
*PUN 105160 B
100/100
*PUN 105182 B
pun 103903
pun 105607
pun 104071
82/81
MAL 80764 C
MAL 105223 C
MIN 101082 C
63/64
MIN 103874 C
PUN 100937 C
PUN 104059 C
PUN 105137 C
PUN 105158 C
*PUN 105160 C
*PUN 105182 C
eic 101422
eic 105159
eic 105407
92/99
rhi 103410
rhi 105448
eic 81803
off 105085
off 106519
off 7904
O. granulata
0.01

GPA1

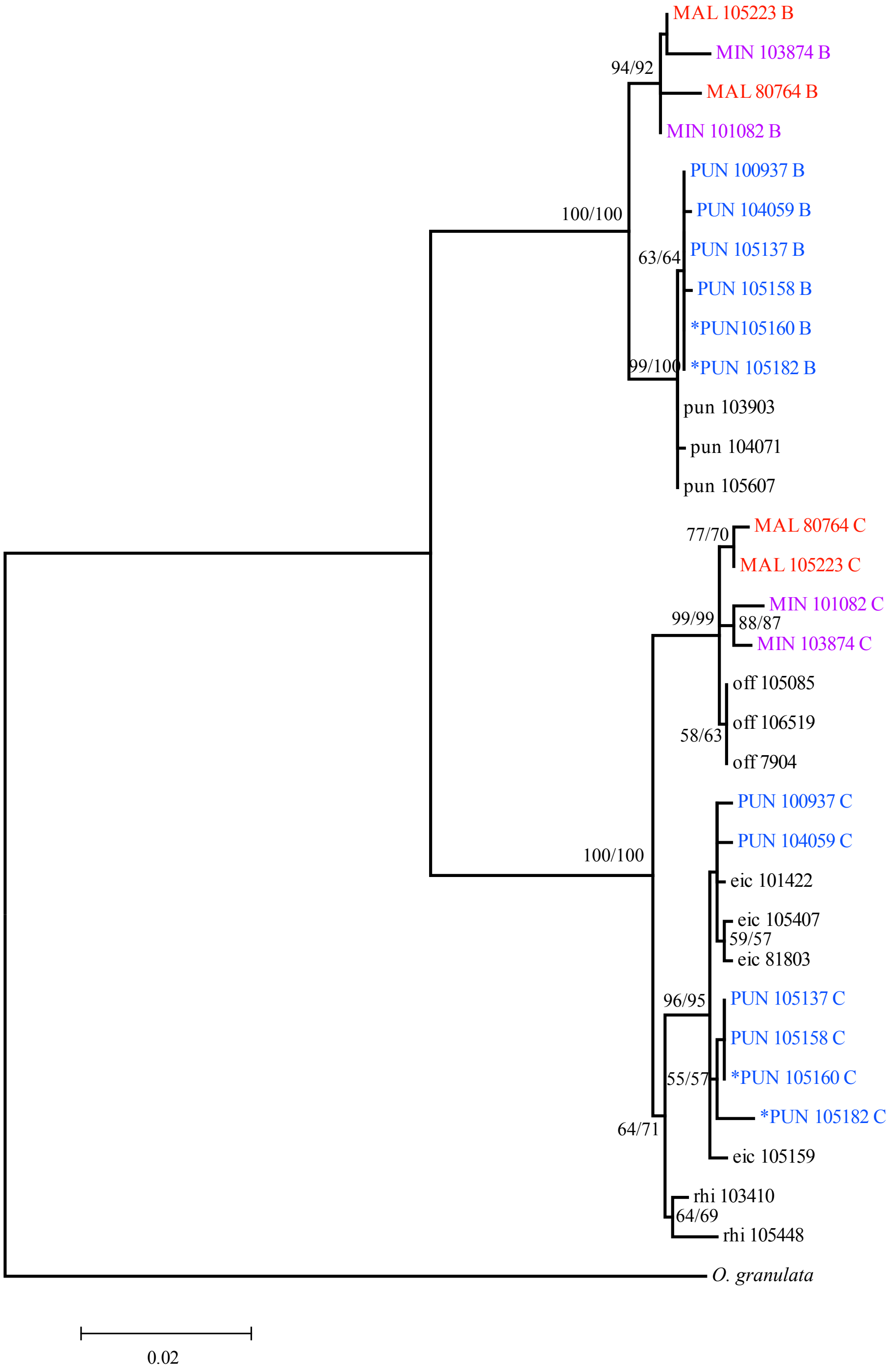
MAL 105223 B
MIN 103874 B
MAL 80764 B
MIN 101082 B
PUN 100937 B
PUN 104059 B
PUN 105137 B
PUN 105158 B
*PUN105160 B
*PUN 105182 B
pun 103903
pun 104071
pun 105607
MAL 80764 C
MAL 105223 C
MIN 101082 C
MIN 103874 C
off 105085
off 106519
off 7904
PUN 100937 C
PUN 104059 C
eic 101422
eic 105407
eic 81803
PUN 105137 C
PUN 105158 C
*PUN 105160 C
*PUN 105182 C
eic 105159
rhi 103410
rhi 105448
O. granulata
94/92
100/100
63/64
99/100
77/70
99/99
88/87
58/63
100/100
59/57
96/95
55/57
64/71
64/69
0.02

LHS1

Supplementary Figure S2. Chronogram of the *Oryza* species with the B-, C- and BC-genome types based on four nuclear loci using relaxed clock implemented in MCMCtree. Branch lengths indicate the posterior means of date estimates with the blue bars representing 95% highest posterior density (HPD) intervals for the divergence times estimates. Nodes of interest are numbered as in figure 4.

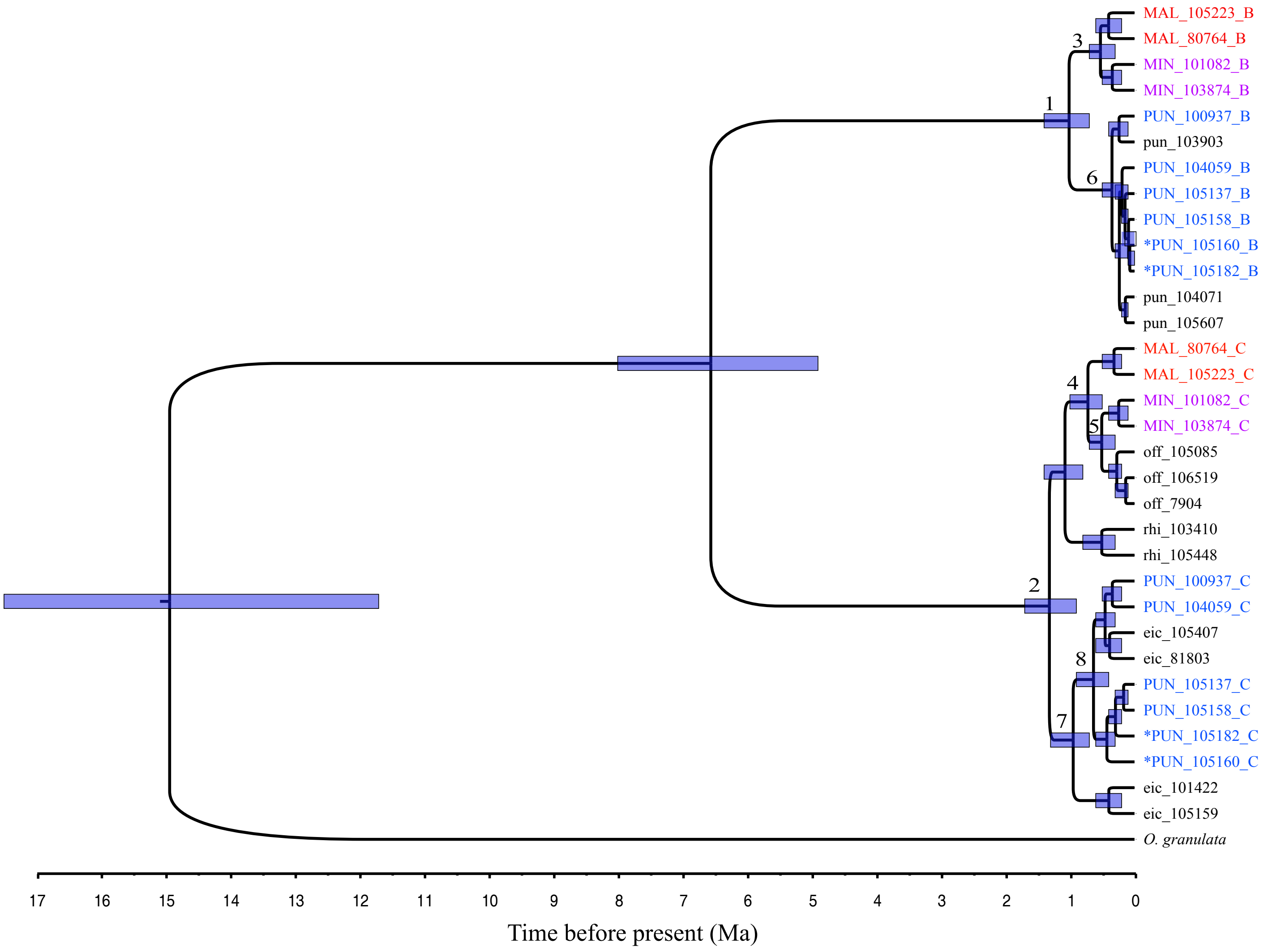

Supplementary Table S1. DNA primers used for amplification and sequencing in this study. Chromosomal locations of nuclear loci are based on rice genome.

**Region**	**Location**	**Primer Sequences (5' to 3')**
Adh1	Chromosome 11	Adh1_F9: ACACCTCTCTTCTTCGGTT<br>Adh1_R9: GTCTTGAACTCGGCATCC<br><br>Adh1_F5: TCCCGTGTTCCCTCGGATCTTC<br>Adh1_R7: ATGCGGATGATGCAGCGGATG<br><br>*Copy-specific primers:*<br>**B copy**<br>Adh1B_2F: CTCTTCTTCGGTTTGTATCCA<br>Adh1B_2R: AAGGAAACATACATACCTGCT<br><br>**C copy**<br>Adh1C_3F: GTGTGGCTTCTCTTGTCTTA<br>Adh1C_1R: GAAAGGAAGGATTCGTGATT
Adh2	Chromosome 11	Adh2B_F: GCTGCAAGAATGGTGAATGG<br>Adh2R_R: CCACCGTTGGTCATCTCAAT<br><br>Adh2_F1: ATCTTYGCAGGGRCAAA<br>Adh2_R1: GATRTACCTCGTGCACT<br><br>*Copy-specific primers:*<br>**B copy**<br>Adh2-Bi2U: ATCAGTGGCTGACTTGGTGT<br>Adh2-Bi7L: GAATTAGTGAGACAGCTGTGATG<br><br>**C copy**<br>Adh2-Ci2F: AGGCCAAGGTAAGCCTCTG<br>(used with Adh2R_R)
GPA1	Chromosome 5	GPA1_15F: TGTGCTTTATGCAAGAGTACGG<br>GPA1_15R: GCACAATGGCTAACTAGAGAAC<br><br>GPA1_F3: CAAATACAATTTAGGTAATCWGC<br>GPA1_R3: AGGATAMAGACCTSAACAGC<br><br>*Copy-specific primers:*

		**B copy**<br>GPA1B_F1: ACAATTTAGGTAATCTGCTG<br>GPA1B_R1: GTGAAACTCTGTTCCTCTCG<br><br>**C copy**<br>GPA1C_1F: GCAAGAGTACGGACAAATGG<br>GPA1C_1R: GGATACAGACCTGAACAGCC
LHS1	Chromosome 3	Lhs1_F2: GCAGCTTGAGAACCAGAT<br>Lhs1_R2: CATGAGGGTGATGATACCTT<br><br>Copy-specific primers:<br>**B copy**<br>LhsF3: ATTCTYGGTGAGGATYTGGGC<br>Lhs1-Bi7R: CCACGGATTATCTACTCGAC<br><br>**C copy**<br>LhsF3: ATTCTYGGTGAGGATYTGGGC<br>Lhs1-Ci7R: CGATGGAGTATATATACAGAACC
matK	chloroplast	matK_F1: TAATTAAGAGGATTCACCAG<br>matK_R1: ATGCAACACCCTGTTCTGAC
trnTD	chloroplast	trnT-52_F: GGTAAGGCATAAGTCATCGG<br>trnD-1181_R: GGTGCTCTGACCAATTGAAC
DEL2	chloroplast	rps16_F: CGTTGCTTTCTACCACATCG<br>trnQ_R: TTACTCGGAGGTTCGAATCC

Supplementary Table S2. Detailed divergence time estimation of the *Oryza* species with the B-, C- and BC-genome types using BEAST and MCMCtree. Numbers are the posterior means of date in millions of years with 95% highest posterior density (HPD) intervals in brackets (only interest nodes in figure 4 are shown).

Nodes in fig. 4	BEAST estimates	MCMCtree estimates
1	0.81 (0.52, 1.13)	1.01 (0.67, 1.35)
2	1.07 (0.76, 1.37)	1.32 (0.92, 1.70)
3	0.46 (0.30, 0.64)	0.53 (0.34, 0.73)
4	0.60 (0.41, 0.81)	0.72 (0.48, 0.96)
5	0.40 (0.25, 0.57)	0.51 (0.32, 0.71)
6	0.32 (0.19, 0.46)	0.35 (0.22, 0.50)
7	0.78 (0.54, 1.05)	0.95 (0.65, 1.26)
8	0.50 (0.33, 0.68)	0.63 (0.42, 0.85)
